# The Cellular and Transcriptomic Early Innate Immune Response to BCG Vaccination in Mice

**DOI:** 10.3390/cells13242043

**Published:** 2024-12-11

**Authors:** Liya G. Kondratyeva, Olga A. Rakitina, Victor V. Pleshkan, Alexey I. Kuzmich, Irina A. Linge, Sofia A. Kondratieva, Eugene V. Snezhkov, Irina V. Alekseenko, Eugene D. Sverdlov

**Affiliations:** 1 Shemyakin-Ovchinnikov Institute of Bioorganic Chemistry of the Russian Academy of Sciences, Moscow 117997, Russia; rakitinaolga97@gmail.com (O.A.R.); vpleshkan@gmail.com (V.V.P.); akrubik@gmail.com (A.I.K.); sofia.a.kondr@gmail.com (S.A.K.); cell6370@yandex.ru (E.V.S.); irina.alekseenko@mail.ru (I.V.A.); 2 National Research Center “Kurchatov Institute”, Moscow 123182, Russia; edsverd@gmail.com; 3 Central Tuberculosis Research Institute, Moscow 107564, Russia; iralinge@gmail.com

**Keywords:** trained immunity, BCG, innate immunity, vaccination, transcriptome, RNA-seq

## Abstract

It is established that BCG vaccination results in the development of both a specific immune response to mycobacterial infections and a nonspecific (heterologous) immune response, designated as trained immunity (TRIM), to other pathogens. We hypothesized that local BCG immunization may induce an early immune response in bone marrow and spleen innate immunity cells. The early transcriptomic response of various populations of innate immune cells, including monocytes, neutrophils, and natural killer (NK) cells, to BCG vaccination was examined. To this end, C57Bl/6J mice were subcutaneously immunized with 10^6^ CFU of BCG. Three days following BCG administration, the three cell populations were collected from the control and BCG-vaccinated groups using FACS. All cell populations obtained were utilized for the preparation and sequencing of RNA-seq libraries. The analysis of FACS data revealed an increase in the proportion of splenic NK cells and monocytes 3 days post-vaccination. Transcriptomic analysis revealed the deregulation of genes associated with the regulation of immune response (according to Gene Ontology terms) in NK cells, monocytes, and unsorted bone marrow cells. Two NK cell-specific immune ligands (Tnfsf14 and S100a8) and two bone marrow-specific immune receptors (C5ar1 and Csf2rb) were identified among differentially expressed genes. No alterations were identified in neutrophils in either their percentage or at the transcriptomic level. Thus, in this study, we demonstrated that BCG vaccination provides an early increase in the proportion of murine bone marrow and spleen immune cell populations, as well as transcriptomic alterations in monocytes, NK cells, and non-sorted bone marrow cells. This early innate immune response may be beneficial for enhancing TRIM.

## 1. Introduction

Epidemiological studies have demonstrated that *Mycobacterium bovis bacillus Calmette–Guérin* (BCG) vaccination significantly reduces infant mortality, which cannot be solely explained by a decrease in tuberculosis incidence [1]. The capacity of BCG vaccination to provide nonspecific (heterologous) protection against respiratory infections was substantiated by randomized clinical trials in adults [2,3,4]. These and other data indicated that innate immunity and relevant cells, such as monocytes, are involved in this protection [5,6]. The long-lasting functional changes in monocytes are linked to the transcriptional, epigenetic, and metabolic reprogramming of innate immune myeloid cell precursors in individuals vaccinated with BCG [7] and represent what has been termed “trained immunity” (TRIM). There is evidence that TRIM is at least partially responsible for how BCG vaccination confers protection against viral infections [8,9,10]. TRIM results in the enhanced responsiveness of immune cells to secondary stimuli [11], providing protection against reinfection in a T/B-cell-independent manner [12]. It is orchestrated by distinct signaling pathways, metabolic alterations, and epigenetic rewriting in both circulating myeloid cells and bone marrow progenitors [10,13]. In the case of BCG, there is also evidence that TRIM can be developed through NK cells [14]. The growing evidence suggests a mutual interaction between trained and adaptive immunity [15,16,17]. Therefore, BCG induces lasting changes in the immune system, which are linked to the heterologous response to infections, both at the level of trained innate immunity and in terms of heterologous responses of the adaptive immune system [18]. However, the duration of TRIM, as well as the time required for TRIM development, remains unclear. According to some data, TRIM can last for several months to over a year due to the presence of long-lived trained macrophages and/or myeloid precursors in the bone marrow [19]. In the case of listeriosis, TRIM can persist for extended periods, with studies showing protection up to 9 weeks post-training [20]. It was also shown that BCG vaccination induces long-lasting effects on both TRIM and heterologous Th1/Th17 immune responses, lasting up to 1 year [21]. TRIM induced by β-glucan one week before infection confers prolonged protection against lethal Listeria monocytogenes infection [20]. It is noteworthy that TRIM is particularly significant in newborns and infants, whose adaptive immunity is still developing [22].

It is notable that in the majority of studies examining the impact of BCG vaccination on the immune system and TRIM development, the authors estimate the immune response no earlier than 14 days following BCG vaccination, with the earliest timepoint being 7 days post-vaccination [23,24,25,26]. Nevertheless, it has been demonstrated that subcutaneous BCG vaccination results in the distribution of bacteria to the lymph nodes (both axillary and inguinal, 100% of mice), lungs (70% of mice), and spleen (30% of mice) as early as 3 days following vaccination [27]. The presence of bacteria in these tissues is likely to exert an influence on the murine immune system. However, this phenomenon has not been previously studied. For a deeper understanding of the early immune response to BCG vaccination and, ultimately, the mechanisms leading to TRIM, it is also important to identify the initial molecular and cellular events that trigger the reprogramming of innate immune cells. In this study, we aimed to evaluate the early response of innate immunity cells to subcutaneous BCG vaccination. To this end, we have vaccinated healthy C57Bl/6J mice with either BCG or phosphate-buffered saline (as a control) and analyzed the immune cell compositions of the bone marrow and spleen, as well as the transcriptomic changes in neutrophils, monocytes, and NK cells. The findings obtained within our study can provide deeper insight into the mechanisms underlying the development of trained immunity.

## 2. Materials and Methods

### 2.1. Animals and Vaccination Procedure

C57Bl/6JCit mice (female, 6–8-weeks old, hereinafter referred to as C57Bl/6J) were used throughout the study. C57Bl/6J mice were bred and maintained under conventional conditions with water and food provided ad libitum at the Animal Facilities of the Central Tuberculosis Research Institute (Moscow, Russia), according to the guidelines of the Russian Ministry of Health, National Institutes of Health Office of Laboratory Animal Welfare (OLAW). The studies using mice were reviewed and approved by the Animal Care and Use Committee of the Central Institute for Tuberculosis, Moscow, Russia (protocol 1 from 3 March 2022). All animal manipulations were performed according to the recommendations of the European Convention for the Protection of Vertebrate Animals Used for Experimental and Other Scientific Purposes, Council of Europe (ETS 123).

A *Mycobacterium bovis bacillus Calmette–Guérin* (BCG) culture, Russian strain, (Medgamal, Moscow, Russia) was used for the vaccination of the animals. Two weeks prior to its administration to the experimental animals, BCG was cultivated in Middlebrook 7H9 Broth (Millipore Sigma, Gillingham, UK) liquid medium with Middlebrook ADC Growth Supplement (HiMedia Laboratories Pvt. Limited, Mumbai, Maharashtra, India) in an incubator at 37 °C. To determine the concentration of mycobacteria in the culture, a series of fivefold dilutions of the suspension was applied as 20 µL drops to Petri dishes with Dubos agar (Dubos, BD Difco, Franklin Lakes, NJ, USA). After the drops dried, the Petri dishes were covered and incubated at 37 °C. After 3–4 days of incubation, the cultures were microscopically examined at ×20 magnification in an inverted microscope, the smallest dilution with non-adherent BCG microcolonies was selected, and at least 500 microcolonies were counted with a total area of counted fields of view ≥ 5% of the total area of the dried drop. The concentration of mycobacteria in the filtrate was expressed as number of colony-forming units per ml (CFU/mL) and calculated using the following formula: CFU/mL = N × (D2/d2/n) × (1000/V) × F. In this equation, *n* is the number of counted microcolonies in all fields of view, D is the diameter of the dried dilution drop in mm, d is the diameter of the field of view in mm, *n* is the number of fields of view, V is the initial drop volume in µL, and F is the dilution factor. After determining the mycobacterial concentration, the BCG cultures were resuspended in PBS at a concentration of 10^7^ CFU/mL. A single BCG stock was utilized for the vaccination of all mice within BCG group, and the microcolonies exhibited no discernible phenotypic differences.

The mice were subcutaneously vaccinated into the right flank with 10^6^ CFU of BCG in 100 µL of phosphate-buffered saline (PBS), and the mice in the control group were subcutaneously injected with 100 µL of PBS (*n* = 5 for each group). Three days following BCG administration, the vaccinated and control animals were sacrificed under isoflurane anesthesia via exsanguination and subsequent cervical dislocation, after which the spleen and bone marrow were collected for further analysis. Downstream cell isolation for each tissue proceeded immediately.

### 2.2. Cell Suspension Preparation

To obtain cell suspensions from the spleen and bone marrow, the organs were washed with 5 mL PBS, homogenized with a flat pestle, passed through a cell filter (70 μm, Cell Strainer), and centrifuged at 250× *g* and 4 °C for 7 min. To remove erythrocytes, the cell pellet was resuspended in 1 mL Red Blood Cell Lysing Buffer Hybri-Max (Sigma–Aldrich, St. Louis, MO, USA) and incubated for 1 min at room temperature, after which 14 mL PBS was added. Cells were resuspended and centrifuged at 250× *g* and 4 °C for 7 min. The pellet was then resuspended in 5 mL of cold PBS and centrifuged again at 300× *g* 4 °C for 5 min. The cells were resuspended in 1 mL of cold PBS, after which the number of cells in the resulting suspension was determined through Trypan Blue staining using the automatic cell counter Countess II FL (Invitrogen; Thermo Fisher Scientific Inc., Waltham, MA, USA). For further staining and FACS, 1 million bone marrow cells and 1 million spleen cells were resuspended in FACS buffer (1% fetal bovine serum and 2 mM EDTA in PBS). Additionally, 1 million unsorted bone marrow cells were collected.

### 2.3. Antibody Staining, Flow Cytometry, and Cell Sorting

In order to exclude nonspecific cell staining, we first incubated the cell suspensions from both bone marrow and spleen tissues with blocking anti-mouse CD16/CD32 antibodies (Mouse BD Fc Block™, Pharmingen, San Diego, CA, USA) for 10 min at room temperature, and then with conjugated antibodies against CD45 (PerCP/Cy5.5 dye, Cat. No. 103132 Biolegend, San Diego, CA, USA), CD11b (FITC dye, Cat. No. 101217, Biolegend, USA), NK1.1 (PE dye, Cat. No. 156504, Biolegend), Ly6G (APC dye, Cat. No. 127613, Biolegend), and Ly6C (APC/Cyanine7 dye, Cat. No. 128025, Biolegend) on ice, in the dark, for 30 min. To exclude the dead cells, we also stained the cell suspensions with DAPI (Bio-Rad, Hercules, CA, USA). In addition to this, Fluorescence Minus One (FMO) controls for each antibody used were prepared for better gating during cell sorting. Following staining, the cells were washed twice with FACS buffer (4 °C, 500× *g*, 7 min) and filtered through a 40 µm Cell Strainer.

Cell population analysis and FACS were performed using a BD FACSAria III (BD Biosciences, Franklin Lakes, NJ, USA) sorter, BD FACSDiva v9.0 (BD Biosciences, Franklin Lakes, NJ, USA) software, and Flowing Software v2.5.1 (University of Turku, Turku, Finland). The parameters of the cell sorter were first adjusted according to the sample staining and FMO controls. After the adjustment of the parameters, the gates containing the target cell populations (monocytes: CD45+CD11b+Ly6C+; neutrophils: CD45+CD11b+Ly6G+; NK cells: CD45+CD11b-NK1.1+) were set. The percentages of the target populations were evaluated for each mouse (*n* = 5 per group). To estimate the statistical significance of the differences between populations from the control and BCG-vaccinated mice, the Mann–Whitney test was used. *p*-values < 0.05 were considered significant.

For both the control and BCG-vaccinated groups of C57Bl/6J mice, cell suspensions from 5 bone marrow and 5 spleen samples were sorted. The maximum available number of neutrophils (300,000 cells) and monocytes (50,000 cells) was sorted from bone marrow samples, given their higher abundance in bone marrow compared to the spleen. Similarly, the 50,000 NK cells were isolated from spleen samples. Due to technical reasons (sample contamination with erythrocytes, interfering with sorting, poor RNA quality with low RIN scores, etc.) and to maintain the pairing of bone marrow and spleen samples, some samples were excluded from further processing. Finally, samples obtained from 3 mice from each group were chosen and further processed for RNA isolation and sequencing.

### 2.4. RNA Isolation and Sequencing

**Figure 1:**
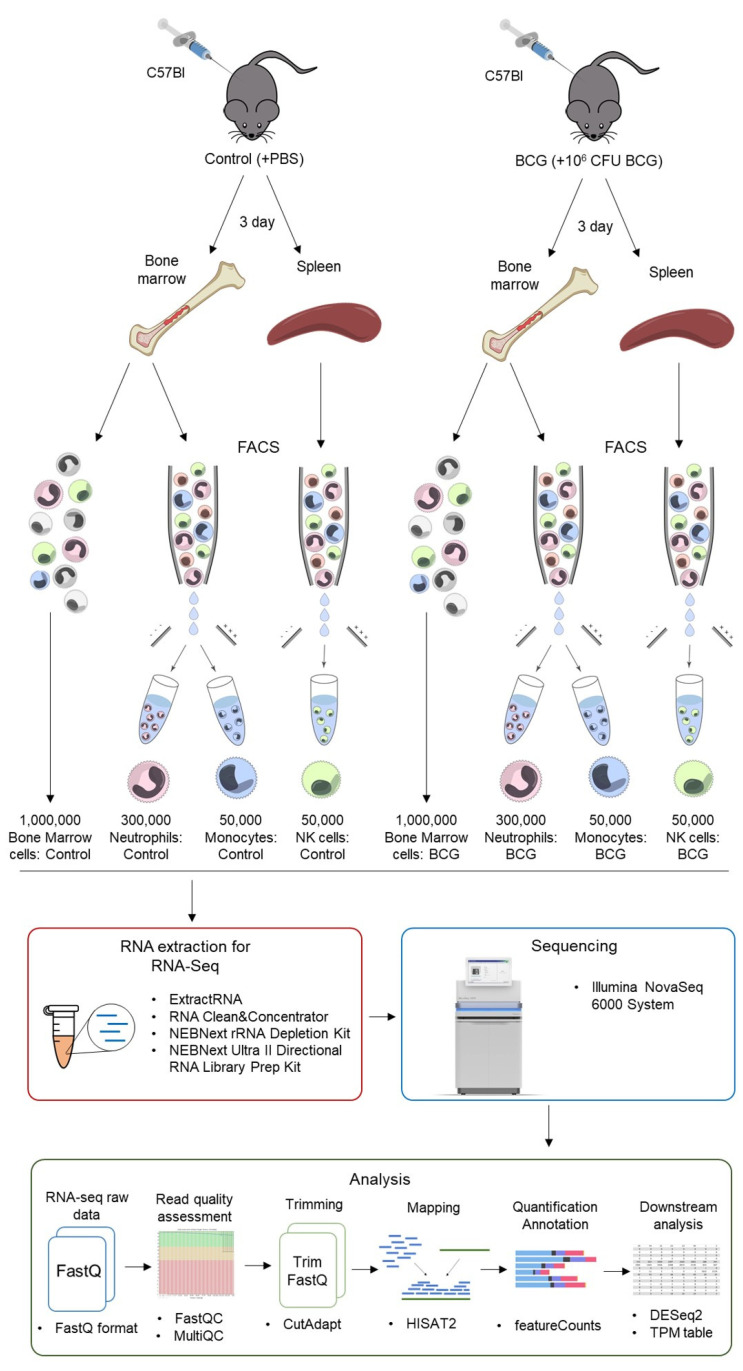
RNA-Seq experiment and sequencing analysis workflow.

The workflow of the preparation of samples for RNA sequencing and further data analysis is presented in Figure 1. The 300,000 neutrophils, 50,000 monocytes, and 50,000 NK cells sorted, as well as 1 million unsorted bone marrow cells of each mouse, were collected via centrifugation and lysed using ExtractRNA reagent (Evrogen, Moscow, Russia). The cell lysates were stored at −70 °C until RNA extraction. RNA was extracted with RNA Clean & Concentrator^TM^-5 (Zymo Research, Irvine, CA, USA). DNase I treatment was performed in column during the extraction. The total RNA was quantified using a Qubit RNA HS Assay Kit (Thermo Fisher Scientific, Waltham, MA, USA) and Invitrogen Qubit Fluorometer (Thermo Fisher Scientific, Waltham, MA, USA). The quality of the extracted RNA was determined using TapeStation 2200 (Agilent Technologies, Santa Clara, CA, USA) with Agilent High Sensitivity RNA ScreenTape (Agilent Technologies). rRNA depletion was performed using the NEBNext rRNA Depletion Kit v2 (Human/Mouse/Rat) with RNA Sample Purification Beads (New England BioLabs, Ipswich, MA, USA). The NEBNext Ultra II Directional RNA Library Prep Kit for Illumina with Sample Purification Beads (New England BioLabs) and a NEBNext Multiplex Oligos for Illumina (Unique Dual Index UMI Adaptors RNA Set 1, New England BioLabs) were used for the preparation of libraries.

Finally, the resulting 24 individually indexed RNA-seq libraries (3 × bone marrow control, 3 × bone marrow BCG, 3 × neutrophil control, 3 × neutrophil BCG, 3 × monocyte control, 3 × monocyte BCG, 3 × NK control, and 3 × NK BCG) were mixed in equimolar amounts, and the final mixture was analyzed with Agilent High Sensitivity DNA ScreenTape (Agilent Technologies). The median fragment length of the pooled fragments was 357 b.p., distributed between 200 and 700 b.p. The sequencing was performed using an Illumina NovaSeq 6000 System (Illumina, San Diego, CA, USA, SP-type, paired-end reads, 150 b.p.).

### 2.5. RNA-Seq Data Analysis

The sequencing data was uploaded to the Galaxy web platform, and an analysis of the data was conducted using the public server available at usegalaxy.org. The quality of raw reads of 24 samples was assessed using the MultiQC tool [28]. The reads were trimmed using the CutAdapt tool [29]. The reads were further aligned to a murine reference genome (GRCm38/mm10) via HISAT2 [30], counted using the featureCount tool [31] (GENCODE vM23, GRCm38.p6 was used as the reference transcriptome), and normalized with DESeq2 [32]. Raw sequencing data and DESeq2-normalized counts have been deposited in NCBI’s Gene Expression Omnibus [33] and are accessible through GEO Series accession number GSE261448 (https://www.ncbi.nlm.nih.gov/geo/query/acc.cgi?acc=GSE261448, accessed on 30 October 2024). The full description of the data is also available at [34].

The pathway enrichment analysis was performed using the Metascape web platform [35] and visualized with REVIGO [36]. Ligand–receptor analysis was performed based on the GO terms corresponding to the differentially expressed genes (DEGs, *p*-adj < 0.05; TPM > 5; FC > 1.5 or FC < 0.6).

## 3. Results

### 3.1. The Bone Marrow and Spleen Immune Cell Composition Following Subcutaneous BCG Vaccination

**Figure 2:**
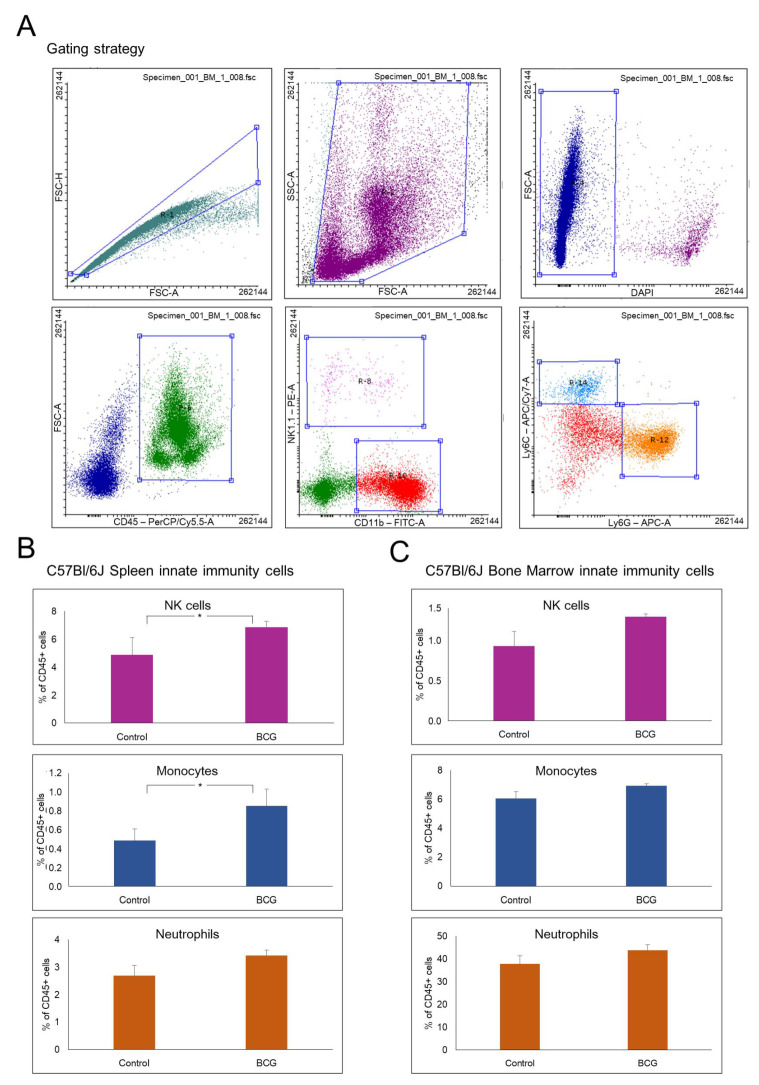
The analysis of murine bone marrow and spleen immune cell populations following BCG vaccination. (**A**) An example of the gating strategy for the bone marrow sample: First, a debris-free and DAPI-negative (live cells) cell population was isolated. Then, among them, a population of leukocytes (CD45+) was distinguished, which was subdivided into two subpopulations: CD45+ CD11b+ (myeloid cells) and CD45+ NK1.1+ (NK cells) immune cells. The population of myeloid cells (CD45+ and CD11b+) was further divided into subpopulations of monocytes (CD45+, CD11b+, and Ly6C+) and neutrophils (CD45+, CD11b+, and Ly6G+). (**B**,**C**) Changes in the proportions of innate immune cell populations in the spleen (**B**) and bone marrow (**C**) 3 days following subcutaneous BCG vaccination. The percentage of neutrophils, monocytes, and NK cells was calculated relative to the total number of leukocytes in the samples. Bars represent the mean among 5 mice per group ± S.D. * *p* < 0.05 (Mann–Whitney test).

The available data on the time required for TRIM development suggests that TRIM is usually induced between 7 and 14 days after the BCG vaccination [20]. We investigated the early changes in the innate immune response of mice 3 days following vaccination, when the pathogen-induced immune response presumably starts evolving into TRIM. This specific timepoint was also chosen since it has been demonstrated that subcutaneous BCG vaccination results in the distribution of bacteria to the lymph nodes (both axillary and inguinal, 100% of mice), lungs (70% of mice), and spleen (30% of mice) as early as 3 days following vaccination [27], and although the presence of bacteria in these tissues is likely to exert an influence on the murine immune system, this phenomenon has not been previously studied. Immunophenotyping of bone marrow and spleen cells was performed 3 days following subcutaneous BCG vaccination. Samples of murine bone marrow and spleen were collected 3 days following subcutaneous BCG injection or subcutaneous PBS injection as a control. The samples were stained using a panel of antibodies that enabled the differentiation of populations of monocytes, neutrophils, and natural killer (NK) cells and analyzed using flow cytometry (the gating strategy is presented in Figure 2A). Briefly, gates for populations of alive, doublet-free, and debris-free cells were initially established. Then, among them, a population of CD45-positive leukocytes was distinguished, which was subdivided into two subpopulations: (i) CD11b-positive myeloid cells and (ii) NK1.1-positive NK cells. The population of myeloid cells was further divided into subpopulations of Ly6C-postitive monocytes and Ly6G-positive neutrophils. As a result, the percentages of NK cells, monocytes, and neutrophils in the bone marrow and spleen 3 days following BCG vaccination were evaluated (Figure 2B,C).

The analysis revealed a slight, although statistically significant, increase in the percentage of splenic monocytes (*p* < 0.05) and NK cells (*p* < 0.05) 3 days following BCG vaccination compared to PBS-injected mice (Figure 2B). In contrast, no statistically significant changes were observed in the percentages of splenic neutrophils and bone marrow innate immune cell populations 3 days following BCG vaccination compared to the control (Figure 2B,C).

### 3.2. The Transcriptomic Response of Innate Immune Cells 3 Days Following BCG Vaccination

To gain a deeper understanding of the impact of BCG vaccination on innate immune cells, we obtained the samples of murine neutrophils, monocytes, and NK cells 3 days following BCG vaccination using FACS and subjected them to RNA sequencing (Figure 1). In order to obtain an adequate number of cells for RNA sequencing, the neutrophils and monocytes were sorted from bone marrow samples, whereas the NK cells were sorted from spleen samples, based on the abundance of these types of cells in the corresponding tissues. The samples of non-sorted bone marrow cells were also collected for each mouse and subjected to RNA sequencing in order to study the general impact of BCG vaccination on bone marrow cells, which include a broad spectrum of immune cell progenitors.

#### 3.2.1. RNA-Seq Data Quality Assessment

In total, 1,418,200,000 double-end 150 b.p. reads were obtained. The fastq files were analyzed using the FASTQC tool [37], and the resulting report indicated an acceptable sequencing quality for all 24 samples. The mean number of paired-end reads per library was 57 million. The quality parameters of the obtained RNA-seq libraries are presented in Table 1.

The reads were subsequently aligned to the murine reference genome (GRCm38/mm10) using the HISAT2 [30] tool. The successfully aligned reads were then counted using the featureCount tool [31] (GENCODE vM23, GRCm38.p6 was used as the reference transcriptome) and normalized with DESeq2 [32]. The obtained read counts were converted into transcripts per million (TPM) values and averaged over the triplicates. The percentage of successfully aligned reads for each library is presented in the Table 1.

The percentages of uniquely mapped and assigned reads in non-sorted bone marrow samples were found to be twofold lower than those observed in the sorted cell populations (Table 1). However, this was an anticipated result based on the sample preparation protocol. The dead cells were specifically excluded during cell sorting based on the DAPI staining, whereas in the case of non-sorted bone marrow cells, there was no step for the exclusion of such cells. The contamination of samples with dead cells may result in a reduction in the number of uniquely mapped reads due to nuclease-mediated mRNA decay, which results in shorter and, therefore, multiple-assigned reads.

To confirm the phenotype of the isolated cell populations (NK cells, neutrophils, and monocytes) and quality of cell sorting, we analyzed the expression levels of genes corresponding to surface markers of the targeted populations, which were used for cell sorting. The results are presented in Supplementary Figure S1. The expression of genes was estimated using the transcripts per million (TPM) method proposed by Galaxy.

As anticipated, the sorted neutrophils and monocytes exhibited high expression levels of the *Ptprc* (pan-leukocyte antigen CD45) and *Itgam* (myeloid cell marker CD11b) genes. The expression levels of their specific markers *Ly6g* (neutrophil marker Ly6G) and *Ly6c1/2* (monocyte marker Ly6C) corresponded with the respective population, thereby confirming the phenotype of the sorted cell populations. The phenotype of the sorted NK cells was also confirmed, since elevated expressions of *Ptprc* and *Klrb1b/c* (NK cell marker NK1.1) were observed.

In the case of non-sorted bone marrow cells, elevated *Ptprc* gene expression was observed. Since the bone marrow contains heterogenous populations of hematopoietic and nonhematopoietic cells, the observed expression level of *Ptprc* was relatively low compared to the sorted cell populations. The cellular composition of bone marrow includes a variety of myeloid cells. This was corroborated by the expression of the *Itgam* gene. Additionally, the bone marrow transcriptomes exhibited the expression of *Ly6g*, *Ly6c1*, and Ly6c2 genes, which correspond to monocyte and neutrophil populations. In turn, the observed *Klrb1b/c* gene expression was low, which can be attributed to the migration of mature NK cells outside the bone marrow.

**Figure 3:**
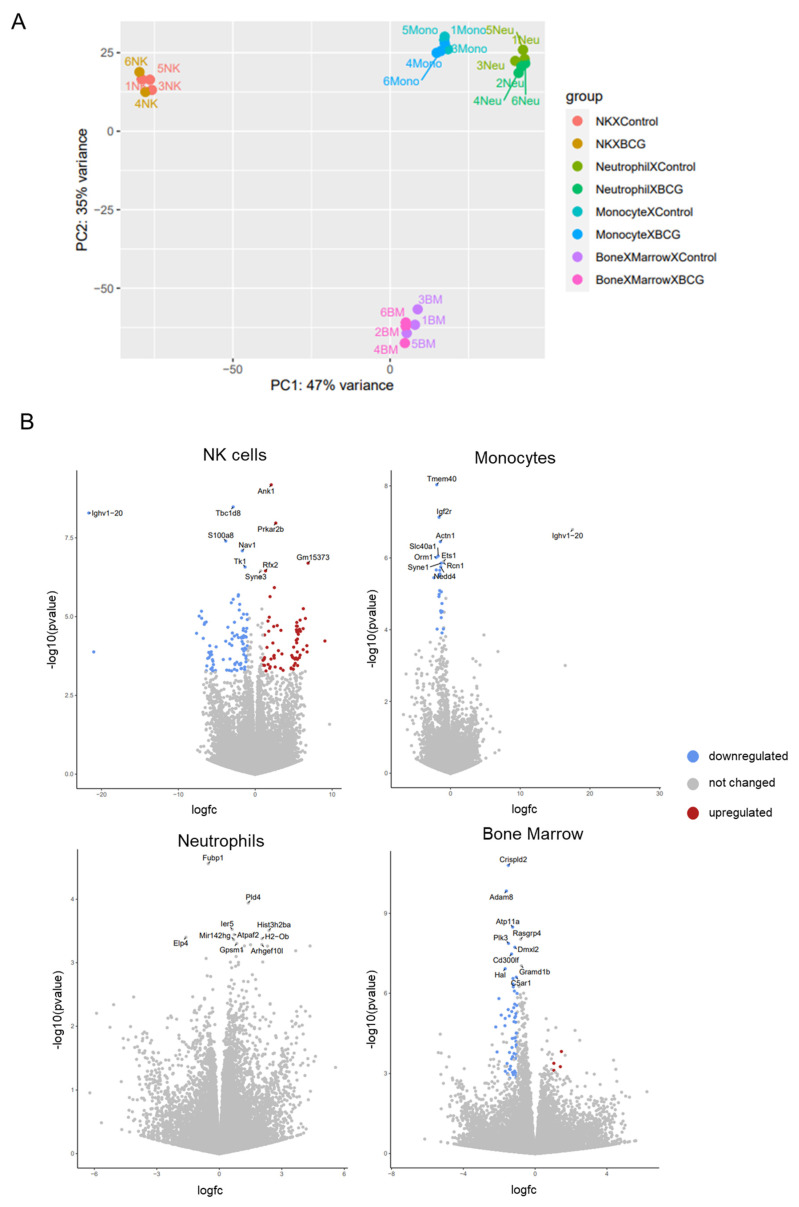
The analysis of transcriptomic data using DESeq2. (**A**) Principal component analysis (PCA) of the normalized RNAseq data of innate immune cells in response to subcutaneous administration of BCG. (**B**) Volcano plots of DESeq2 results based on RNA-seq analysis of BCG-induced innate immune cells over control. Changes in transcript levels are represented by log2fc, the log2 fold change in normalized read counts between the immune populations derived from BCG-vaccinated and control mice, as obtained from the DESeq2 analysis [32] (*x*-axis). The statistical significance of the change is represented as −log10 *p*-adj (*y*-axis). Differentially expressed genes (DEGs) are highlighted in red (upregulated) and blue (downregulated) with significant adjusted *p*-values (*p*-adj < 0.05).

In order to examine the inter-relations among populations, we plotted all samples in a two-dimensional principal component analysis (PCA) plot based on their gene expression levels (Figure 3A). PCA analysis revealed general differences between groups of samples: PC1 divided the samples by sub-type, with myeloid monocytes and neutrophils being clearly separated from lymphoid NK cells. At the same time, PC2 clearly separated non-sorted bone marrow and sorted cell populations.

To identify differentially expressed genes (DEGs) between BCG-induced and control cells of each population, we defined genes with an adjusted *p*-value of less than 0.05 and fold change of higher than 2 as DEGs (Figure 3B, Supplementary Table S1). Using the DESeq2 approach [32], a total of 63 DEGs (3 upregulated and 60 downregulated) were identified in BCG-induced unsorted murine bone marrow cells compared to control non-vaccinated mice; 26 downregulated genes were observed in BCG-induced monocytes (with no upregulated genes); 70 upregulated and 92 downregulated genes were identified in BCG-induced NK cells. The gene expression profile of neutrophils did not differ between BCG-vaccinated and control mice. Thus, NK cells, monocytes, and non-sorted bone marrow cells exhibited transcriptomic alterations as early as three days following vaccination. Subsequently, we sought to identify the specific genes that were involved in this response.

#### 3.2.2. Gene Ontology (GO) and Pathway Enrichment Analyses

The identified differently expressed genes (DEGs) from bone marrow, monocyte, and NK cell samples were subjected to Gene Ontology (GO) function and pathway enrichment analyses using Metascape [35].

#### 3.2.3. Transcriptomic Alterations in NK Cells

**Figure 4:**
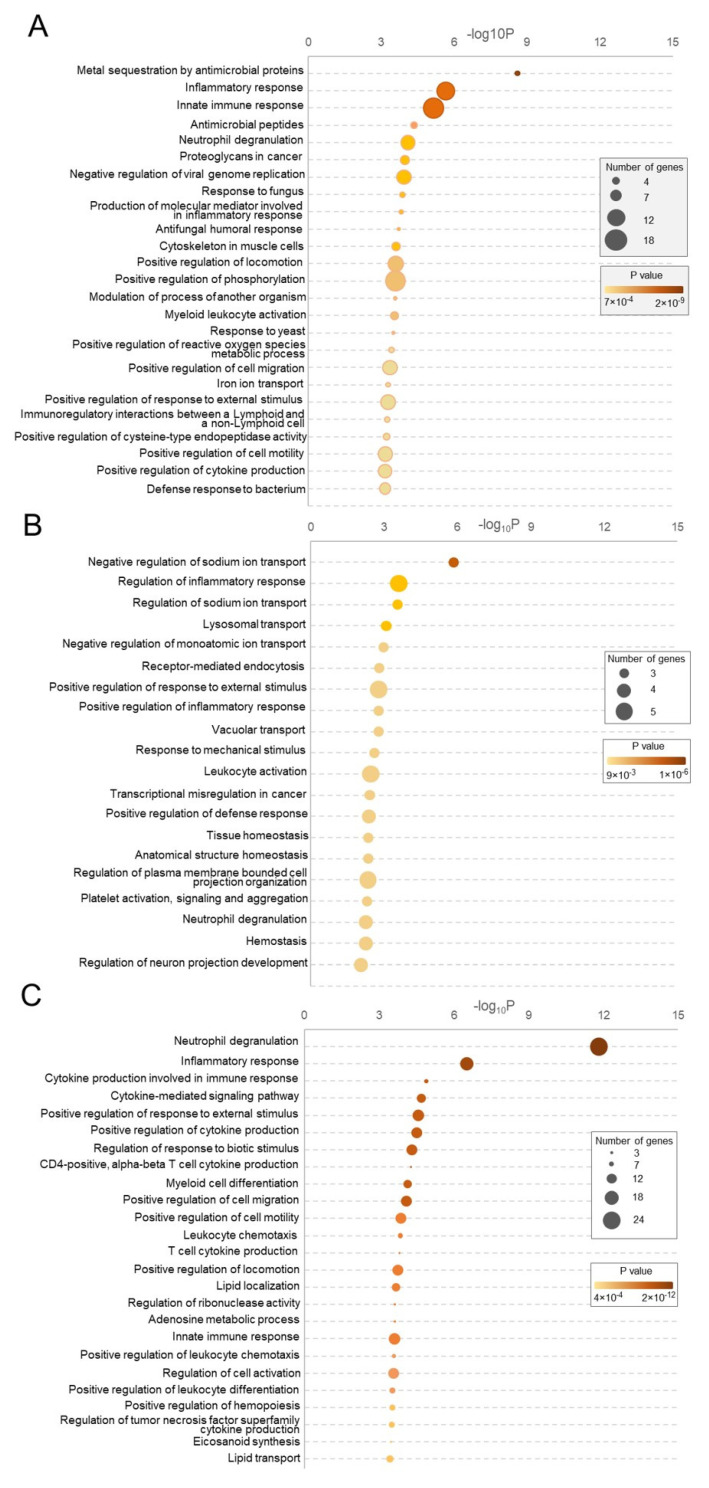
The functional annotation of the revealed DEGs. (**A**) The functional annotation of 162 DEGs from splenic NK cells from mice after BCG vaccination was conducted using Metascape web-platform, resulting in the representation of the top 25 terms in a bar plot based on their *p*-value (log10 scale). (**B**) The functional annotation of 30 DEGs from Bone Marrow Monocytes from mice after BCG vaccination was conducted using Metascape web-platform, resulting in the representation of overall 20 terms in a bar plot based on their *p*-value (log10 scale). (**C**) The functional annotation of 184 DEGs from bulk Bone Marrow cells from mice after BCG vaccination was conducted using Metascape web-platform, resulting in the representation of top 25 terms in a bar plot based on their *p*-value (log10 scale).

To determine early changed processes and pathways in NK cells after BCG vaccination, we analyzed 179 DEGs (*p*-adjusted < 0.05, FC > 1.5 and FC < 0.6). The top 25 GO terms that were enriched in NK cells upon BCG vaccination are represented in Figure 4A. Using Metascape, the subclass of representative terms from the gene function analysis was transformed into a network arrangement (Supplementary Figure S2A). The complete list of enriched GO terms and corresponding enrichment scores are presented in Supplementary Table S1.

The results demonstrated a significant enrichment in GO terms associated with the regulation of the immunological response upon BCG vaccination, including “inflammatory response”, “innate immune response”, “antimicrobial peptides”, “neutrophil degranulation”, “myeloid leukocyte activation”, “antifungal humoral response”, “response to fungus”, “response to yeast”, “defense response to bacterium”, “immunoregulatory interactions between a lymphoid and a non-lymphoid cell”, “NF-kappa B signaling pathway”, etc. (Figure 4A, Supplementary Figure S2A). Another set of enriched GO terms was associated with metabolic and molecular processes: “production of molecular mediator involved in inflammatory response”, “positive regulation of reactive oxygen species metabolic process”, “iron ion transport”, “positive regulation of cytokine production”, “positive regulation of cysteine-type endopeptidase activity”, etc. (Figure 4A, Supplementary Figure S2A). The enrichment of general cellular processes was also observed, including “cytoskeleton in muscle cells”, “positive regulation of phosphorylation”, “positive regulation of cell migration”, “positive regulation of cell motility”, “positive regulation of locomotion”, “modulation of process of another organism”, “positive regulation of response to external stimulus”, etc. The top five deregulated genes included three downregulated—*Fzd4*, *Camp*, and *Ank2*—and two upregulated—*Necab2* and *Kncn*—genes. We have additionally evaluated the expression of NK cell proliferation/activation marker genes, including *Ifng*, *Cd69*, *Gzmb*, and *Mki67*; however, no change was observed in their expression.

These results indicate the involvement of genes whose expression was altered in NK cells upon BCG vaccination in metabolic, molecular, and cellular processes, aligning with the mechanisms underlying the development of TRIM at later timepoints.

#### 3.2.4. Transcriptomic Alterations in Monocytes

To determine early changed processes and pathways in monocytes following BCG vaccination, we analyzed all 30 downregulated genes (*p*-adjusted < 0.05, FC < 0.6). The top GO terms that were enriched in monocytes upon BCG vaccination are represented in Figure 4B, and the network analysis of the subclasses of these terms is represented in Supplementary Figure S2B.

The analysis revealed a significant enrichment in GO terms related to various processes involved in the regulation of the immune response upon BCG vaccination, including “regulation of inflammatory response”, “positive regulation of inflammatory response”, “leukocyte activation”, “positive regulation of defense response”, “neutrophil degranulation”, “cellular transport and endocytosis”, “lysosomal transport”, “vacuolar transport”, “receptor-mediated endocytosis”, “cellular sensing and response”, “response to mechanical stimulus”, “positive regulation of response to external stimulus”, “cellular homeostasis and structure”, “tissue homeostasis”, “anatomical structure homeostasis”, “platelet activation and hemostasis”, “platelet activation, signaling, and aggregation”, and “hemostasis”. The top five down-regulated genes included *Enpp5*, *Orm1*, *Atp8a2*, *Tmem40*, and *Stk39*. Their potential role is addressed in the Discussion section.

#### 3.2.5. Transcriptomic Alterations in the Non-Sorted Bone Marrow

To evaluate early changed processes and pathways in bulk bone marrow cells after BCG vaccination, we analyzed 184 DEGs (19 upregulated and 165 downregulated, *p*-adjusted < 0.05, FC < 0.6 and FC > 1.5). The top GO terms that were enriched in the bone marrow cells upon BCG vaccination are represented in Figure 4C, and the network analysis of the subclasses of these terms is represented in Supplementary Figure S2C.

The enriched-upon-vaccination GO terms can be subdivided into four categories. The first category, similar to NK cells and monocytes, was associated with immune system-related processes. It can be further subdivided into two categories, with the first being inflammatory response and cytokine regulation (“inflammatory response”, “cytokine production involved in immune response”, “cytokine-mediated signaling pathway”, “positive regulation of cytokine production”, etc.) and the second being immune response regulation (“regulation of leukocyte activation”, “regulation of immune response”, “negative regulation of immune response”, “regulation of T cell activation”). The second category included GO terms associated with cell migration and chemotaxis, including “positive regulation of cell migration”, “positive regulation of leukocyte chemotaxis”, “positive regulation of chemotaxis”, “regulation of leukocyte migration”, etc. The third category was comprised of GO terms united by cellular activation and differentiation processes, such as “positive regulation of cell activation”, “positive regulation of leukocyte differentiation”, “regulation of myeloid cell differentiation”, and “myeloid leukocyte differentiation”. Finally, the last category was associated with metabolic processes and signaling pathways: “adenosine and purine metabolic processes”, “insulin signaling pathway”, “toll-like receptor signaling pathway”, ”glycerolipid and lipid metabolism”, etc. The top five deregulated genes included five downregulated genes: *Fosl1*, *Trem1*, *Sema6b*, *Hal*, and *Adam8*.

The analysis of the identified categories demonstrated a significant association between the deregulated processes and the immune system. Many of these processes, such as “inflammatory response”, “cytokine regulation”, “cell migration”, and “cellular activation”, are directly linked to immune functions and TRIM development. Additionally, the observed regulation of metabolic pathways and signaling cascades indicates a coordinated early response of bone marrow to the administration of BCG, suggesting a proactive engagement of the immune system in the defense against external stimuli. The intricate interplay of these processes highlights the dynamic nature of immune activation and underscores the comprehensive immune-modulatory mechanisms involved in response to immunological triggers such as BCG.

#### 3.2.6. Analysis of Common Altered Processes

**Figure 5:**
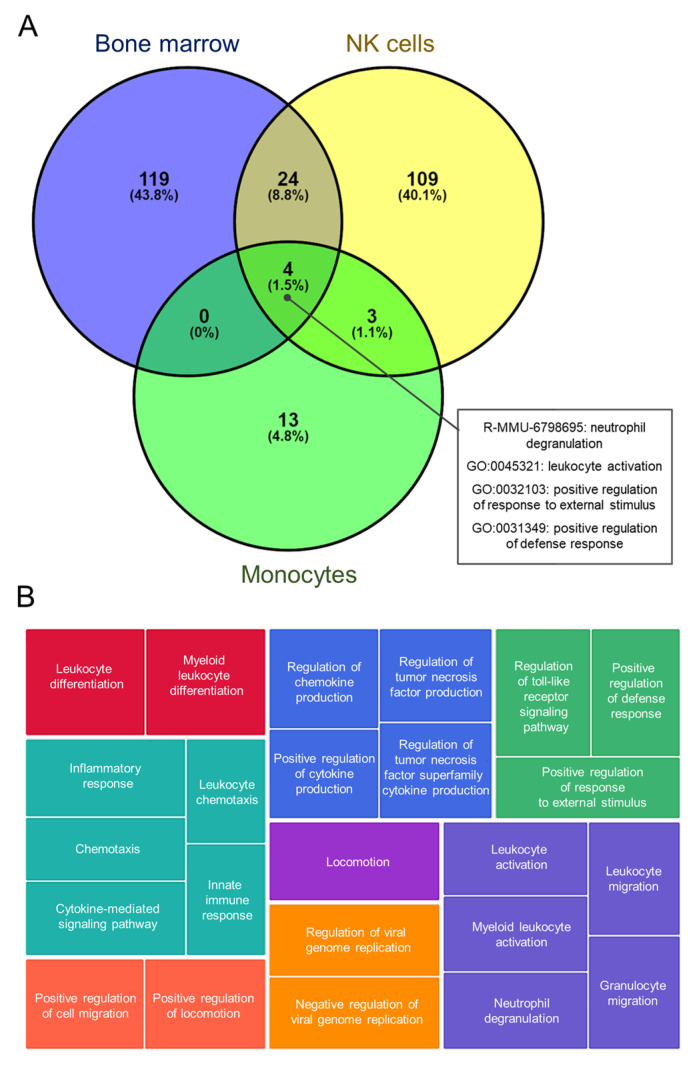
The analysis of common altered processes. (**A**) Venn diagram represents common processes found for DEGs of NK cells, monocytes, and bone marrow cells after BCG treatment. The percentage in the parenthesis reflects the percentage of processes of the overall number of unique processes among 3 populations. (**B**) TreeMap visualization of clustered common deregulated processes in NK cells, monocytes, and bone marrow cells after BCG treatment.

To gain a more comprehensive understanding of the early transcriptomic responses of different immune cells to BCG vaccination, we compared the enriched GO terms in NK cells, monocytes, and non-sorted bone marrow cells following BCG vaccination. The comparison revealed several common processes (Figure 5A). Four common GO terms were shared between the three cell populations: “neutrophil degranulation”, “leukocyte activation”, ”positive regulation of response to external stimulus”, and “positive regulation of defense response”. Three GO terms were shared between NK cells and monocytes: “positive regulation of inflammatory response”, “regulation of inflammatory response”, and “hemostasis”. Finally, 24 terms were shared between bone marrow and NK cells. These terms were clustered using TreeMap visualization (Figure 5B) from Revigo [36], and the majority of terms were associated with immune response, cytokine signaling, and cell migration (the complete list of common GO terms can be found in Supplementary Table S1).

#### 3.2.7. Immune Ligand and Receptor Analysis

**Figure 6:**
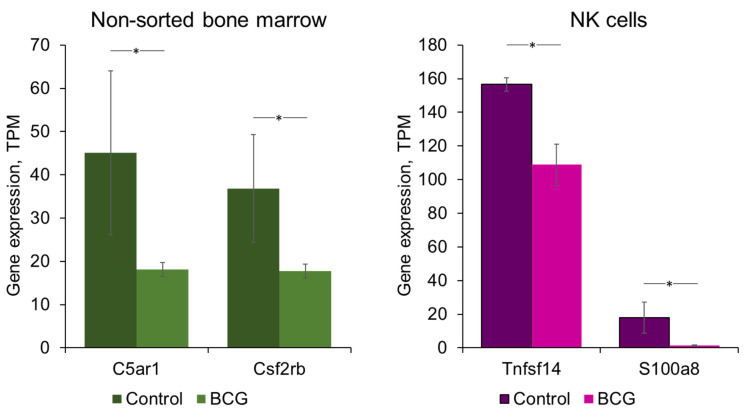
Analysis of immune ligand and receptor gene expression. The expression levels were extracted from the RNA-seq data and presented as the mean TPM (transcripts per million) values with standard deviation bars (*y* axis) calculated from 3 independent samples. Gene abbreviations: C5ar1, complement C5a Receptor 1; Csf2rb, cytokine receptor common subunit beta; Tnfsf14, tumor necrosis factor superfamily member 14; S100a8, S100 calcium-binding protein A8. * *p*-adj < 0.05 according to DeSeq2 data.

The Gene Ontology enrichment analysis revealed that the transcriptomic alterations were predominantly associated with immune response, albeit with considerable ambiguity. To identify the specific genes responsible for these alterations, as well as to better understand the immunity-related processes that occur upon BCG vaccination, we conducted an analysis of immune ligand and receptor gene expression. To this end, we selected genes encoding the surface ligands and receptors associated with immune response from the DEGs (*p*-adj < 0.05; TPM > 5; FC > 1.5 or FC < 0.6) for each population (NK cells, monocytes, and non-sorted bone marrow cells) and analyzed their expression in terms of transcripts per million (TPM). The analysis revealed four genes, two of which were specific to NK cells (*Tnfsf14* and *S100a8*) and two of which were specific to non-sorted bone marrow cells (*C5ar1* and *Csf2rb*), as illustrated in Figure 6. No significantly altered genes encoding immune-related ligands or receptors were revealed for monocytes.

## 4. Discussion

In this study, we evaluated the early response of innate immunity cells to subcutaneous BCG vaccination using the analysis of immune cell population composition and transcriptomic changes in neutrophils, monocytes, and NK cells derived from murine bone marrow and spleen tissues. We chose to evaluate the innate immune response 3 days following vaccination, since BCG vaccination was shown to lead to the distribution of bacteria throughout murine organs as early as 3 days following vaccination [27], but the effect of this distribution on the immune cells has not been previously investigated. To this end, we have subcutaneously immunized C57Bl/6J mice with 10^6^ CFU of BCG or phosphate-buffered saline as a control. Three days following BCG administration, the three cell populations were collected from the control and BCG-vaccinated groups using FACS.

First, we observed a slight, although significant, increase in the percentage of splenic monocytes 3 days following subcutaneous BCG vaccination (Figure 2B). The adult spleen contains a reservoir of monocytes that can be rapidly recruited in response to injury or inflammation [38]. It has been demonstrated that during inflammation, splenic Ly6C-high and Ly6C-low monocytes are mobilized into the circulation via Angiotensin-II/AGTR1A-signaling [39]. The splenic monocytes reside within the red pulp [40]. In rodents, the splenic red pulp also serves as a major site of reactive hematopoiesis, including myelopoiesis [41]. We observed an increase in the percentage of splenic monocytes, which can be indicative of both monocyte–pathogen interactions and the occurring reactive myelopoiesis.

At the same time, the percentage of bone marrow monocytes did not change 3 days following BCG vaccination. Other studies have indicated that systemic (intravenous) BCG vaccination induces a myeloid differentiation bias at the level of hematopoietic stem cells within the bone marrow, resulting in an increased release of monocytes with enhanced cytokine secretion and pathogen-killing abilities several weeks following vaccination [7]. This reprogramming at the progenitor cell level in the bone marrow can result in the emergence of trained monocytes, which can be identified in the circulation of BCG-vaccinated individuals for at least three months post-vaccination [42]. Furthermore, macrophages derived from the bone marrow of BCG-vaccinated mice exhibit an increased bacterial killing capacity for an extended period post-vaccination [43]. This enhanced bacterial killing capacity has been associated with distinct gene expression signatures in macrophages derived from vaccinated mice compared to those from unvaccinated mice [44].

We suggested that while the percentage of bone marrow monocytes might remain the same, we still might detect transcriptomic alterations in these cells; therefore, we obtained samples of monocytes from both BCG-vaccinated and control mice using FACS and subjected them to RNA sequencing. The analysis of RNA-seq data revealed 30 genes, the expression of which was downregulated upon vaccination. Most of the GO terms enriched upon BCG vaccination were associated with the regulation of the immune response (Figure 4B). The top five downregulated genes included *Enpp5*, *Orm1*, *Atp8a2*, *Tmem40*, and *Stk39*. Of these, Enpp5, Atp8a2, and Tmem40 were not previously described in the context of infection, although an Enpp5 paralog, Enpp4, was reported to be induced in BCG-activated macrophages [45]. The Orm1 protein was shown to be responsible for M2b monocyte polarization, leading to decreased antibacterial host immunity [46]; therefore, the downregulation of this gene in the context of BCG vaccination is apparently due to the need for the maintenance of antibacterial host defenses. The SPAK protein, which is encoded by the *Stk39* gene, was shown to be up-regulated in *Mycobacterium marinum* infection of zebrafish embryos 3 days post-infection, and *Stk39* knockdown in this model had no effect on the bacterial burden [47]. At the same time, it was shown that SPAK can play a role in activating macrophage inflammation in both lung injury and inflammatory bowel disease models [48,49]; therefore, the exact role of SPAK might be dependent on the organism and on the type of inflammation. It was also shown that SPAK inhibits potassium influx into the cell, while potassium efflux is a trigger for NLRP3 inflammasome activation [50,51]. The NLRP3 inflammasomes, in turn, are associated with monocyte activation [52]. Therefore, the observed downregulation of *Stk39* upon BCG vaccination might serve as a way of bone marrow monocyte activation.

Overall, the observed downregulation of inflammation-suppressor genes is more likely indicative of bacterial inflammation-related monocyte–pathogen interactions than the occurring reactive myelopoiesis, especially considering the absence of cell division-related gene upregulation. However, further study and confirmation at the protein and functional levels are necessary to substantiate this hypothesis.

In conclusion, while the immediate impact on bone marrow monocyte levels may not be evident shortly after BCG vaccination, the reprogramming of hematopoietic stem cells and progenitor cells in the bone marrow leads to significant changes at the transcriptomic level, affecting the functional capabilities of monocytes in the long term.

Second, we observed a slight increase in the percentage of splenic NK cells 3 days following subcutaneous vaccination (Figure 2B). The spleen, an important secondary lymphoid organ, contains a significant number of NK cells [53,54]. In a study by Dokun et al. [55], the kinetics of the percentage of splenic NK cells during a viral infection in mice was investigated. The authors demonstrated an indiscriminate decrease in the total number of NK cells by day 2 following the infection, with a subsequent increase by day 6. This initial decrease could have been attributed to either cellular migration or cell death.

It is established that BCG vaccination induces rapid and robust NK cell responses in infants. Within 24 to 30 h of stimulation with extracellular BCG, NK cells demonstrate peak IFN-γ production [56]. The vaccination of newborns with BCG results in significantly higher percentages of IFN-γ-expressing NK cells compared to unvaccinated infants at 5 and 9 weeks of age [15,57]. These BCG-reactive NK cell responses correlate with increased levels of secreted cytokines, including IL-2, IFN-γ, IL-6, IL-1β, and TNF [57]. However, the response varies depending on the site and route of vaccine administration. While peritoneal NK cell activity is markedly enhanced by intraperitoneal BCG injection in mice, splenic NK cell activity is suppressed for a period of 5–14 days post-injection [58].

Our results align with these findings, highlighting a pivotal role of NK cells in the early immune response to BCG vaccination. This suggests that NK cells may contribute to protection against tuberculosis and other infections by enhancing innate immune functions.

In order to study the mechanisms underlying the observed changes, we also obtained samples of NK cells from both BCG-vaccinated and control mice using FACS and subjected them to RNA sequencing. The analysis of RNA-seq data revealed 179 genes, the expression of which was de-regulated upon vaccination. Most of the GO terms enriched upon BCG vaccination were associated with the regulation of the immunological response, metabolic and molecular processes, and general cellular processes (Figure 4A). The top five deregulated genes included three downregulated—*Fzd4*, *Camp*, and *Ank2*—and two upregulated—*Necab2* and *Kncn*—genes. Of these, Ank2 was not previously described in the context of infection. The expression of Fzd4 was shown to be elevated in macrophages upon BCG interaction [59]. Moreover, it has been demonstrated that Wnt2b-mediated activation of Fzd4 in NK cells results in WNT/β-catenin pathway activation and enhanced IL33 secretion, thus promoting inflammation [60]. The *Camp* gene encodes multiple antimicrobial peptides [61], however, the role of NK cells in the context of Camp expression has not been studied yet [62]. The observed downregulation of these two genes might indicate the initial immune response suppression. The Necab2 gene was shown to be downregulated in zebrafish larvae upon *Vibrio parahaemolyticus* infection and upregulated upon infection in Notch-knockout larvae. The authors suggested that this gene may function downstream of Notch1a to defend against infection [63]. The observed upregulation of this gene might as well indicate the suppression of initial inflammation by NK cells. Finally, the Kncn protein was shown to bind to *M. tuberculosis* proteins with high affinity [64]. However, the specific function of such binding was not studied. We have additionally evaluated the expression of NK cell proliferation/activation marker genes, including *Ifng*, *Cd69*, *Gzmb*, and *Mki67*, however, no change was observed in their expression, which presumably reflects NK cell recruitment rather than proliferation/activation.

Overall, the observed elevation in the percentage of NK cells combined with the suppression of pro-inflammatory and activation of anti-inflammatory genes 3 days following BCG vaccination suggests the involvement of NK cells in the suppression of initial bacterium-induced inflammation. However, this phenomenon should be further thoroughly studied and confirmed at the protein and functional levels.

Finally, we did not detect immediate changes in the percentages of bone marrow and splenic neutrophils 3 days following BCG vaccination. It was previously demonstrated that neutrophil maturation in murine bone marrow takes 2–3 days, with mature neutrophil lifespan reaching 10–13 h in circulation and 8–18 h at tissue sites under non-inflammatory conditions. It was also demonstrated that under inflammatory conditions, the neutrophil maturation time in the bone marrow and half-life in the blood reduces, and the residence time at the inflammatory site increases [65]. Despite the short half-life of neutrophils, these cells demonstrate BCG-induced trained immunity [66]. Since we aimed to study the early response of cell populations that are important for TRIM development, we suggested that while the percentage of neutrophils might remain the same, we still might detect transcriptomic alterations in these cells. We obtained samples of neutrophils from both BCG-vaccinated and control mice using FACS and subjected them to RNA sequencing. However, the gene expression profile of neutrophils did not differ between BCG-vaccinated and control mice, indicating that neutrophil training more probably occurs at the progenitor level.

We also investigated bulk bone marrow samples of BCG-vaccinated and control mice at the transcriptomic level in order to study the general impact of BCG vaccination on bone marrow cells, which include a broad spectrum of immune cell progenitors. The analysis of RNA-seq data revealed 184 genes, the expression of which was de-regulated upon vaccination. Most of the GO terms enriched upon BCG vaccination were associated with immune system processes (inflammatory response and cytokine regulation; immune response regulation), cell migration and chemotaxis, cellular activation and differentiation metabolic processes, and signaling pathways (Figure 4C). The top five deregulated genes included five downregulated genes: *Fosl1*, *Trem1*, *Sema6b*, *Hal*, and *Adam8*. The Fosl1 protein was shown to be upregulated in trained human monocytes 3 months following BCG vaccination [67], as well as in gastric cells following Helicobacter pylori infection [68]. In the case of *Trem*, it was demonstrated that the expression of this gene is elevated in circulating neutrophils during respiratory BCG infection, and the knockdown of this gene resulted in elevated bacterial loads [69]. However, in this study, we observed the downregulated expression of these genes in bone marrow cells, which might indicate the suppression of an initial immune response. The function of Sema6b in infection is poorly studied, and the available data only focuses on the relevance of this receptor in sensitivity to the *Paeniclostridium (Clostridium) sordelii* pathogen [70]. The host Hal protein was shown to be upregulated 2 weeks following *M. tuberculosis* infection, possibly to starve the bacteria of free intracellular histidine [71]. In our study, we observed that 3 days following BCG vaccination, the expression of this gene is downregulated, which indicates the need to further study the kinetics of this gene in mycobacterial infection. Finally, Adam8 metalloproteinase was shown to be responsible for pathological pneumonia-related neutrophil infiltration of the lungs [72].

Overall, the observed suppression of pro-inflammatory genes 3 days following BCG vaccination suggests the involvement of bone marrow cells in the suppression of initial bacterium-induced inflammation. However, further study and confirmation at the protein and functional levels are necessary to substantiate this hypothesis, especially considering the poor quality of sequencing data in the bone marrow samples.

The analysis of the identified GO categories demonstrated a significant association between the deregulated processes and the immune system. The observed regulation of metabolic pathways and signaling cascades in bone marrow indicates a coordinated early response to the administration of BCG, suggesting a proactive engagement of the immune system in the defense against external stimuli. The intricate interplay of these processes highlights the dynamic nature of immune activation and underscores the comprehensive immune-modulatory mechanisms involved in the response to immunological triggers such as BCG.

Finally, we conducted an analysis of the expression of immune ligand and receptor genes to identify the specific genes that may be responsible for the corresponding transcriptomic alterations. The analysis identified the most significantly altered genes for further examination. Four genes were revealed: two specific to NK cells (Tnfsf14 and S100a8) and two specific to non-sorted bone marrow cells (C5ar1 and Csf2rb), as shown in Figure 6.

*Tnfsf14*, also designated as LIGHT, exhibits a paradoxical function with respect to inflammatory processes and immune responses. Several studies demonstrate that LIGHT enhances inflammatory processes [73,74,75,76]. Increased LIGHT expression has been shown to promote the development of colitis [74], skin inflammation [75], and lung inflammation [76]. Furthermore, LIGHT is predominantly expressed on activated T cells and activated NK cells [77]. Conversely, other studies indicate that LIGHT plays a protective role in the prevention of colitis [78,79].

Accordingly, the observed decline in LIGHT expression three days following BCG vaccination may be attributed to either the attenuation of the initial inflammatory response triggered by vaccination or the promotion of subsequent inflammation. Based on the transcriptomic analysis results, it appears more likely that the former scenario applies in this case. Regarding NK cell-specific LIGHT production, the available data are limited. For instance, the activation of LIGHT production by NK cells has been observed in tumor-sensing NK cells, leading to the priming of novel antitumor responses through the interaction of NK-derived LIGHT with its receptor, HVEM (Tnfrsf14), on the surface of dendritic cells [77]. This interaction accounts for the majority of the immune-stimulating properties of LIGHT. The HVEM receptor is expressed on the surfaces of both NK and T cells, and it serves as an important T-cell costimulatory agent, leading to activation, proliferation, and survival. Another major LIGHT receptor, LTβR, has been demonstrated to play a pivotal role in the development of T-cell-mediated immunity against infection [80].

*S100a8* is a gene that encodes a subunit of the S100A8/A9 pro-inflammatory protein. During the inflammatory process, S100A8/A9 is released actively and exerts a critical role in modulating the inflammatory response by stimulating leukocyte recruitment and inducing cytokine secretion [81]. The dysregulation of S100A8/A9 expression has been associated with severe inflammatory reactions, such as those observed in critical cases of Coronavirus Disease 2019 (COVID-19), especially during the initial stages of infection [82]. Transcriptomic analysis has revealed a notable decline in S100a8 expression in NK cells three days following vaccination, suggesting that the initial inflammatory response may be suppressed by the third day.

Given the observed suppression of inflammation in NK cells three days following subcutaneous BCG vaccination and the effect of LIGHT on T cells, it can be postulated that the suppression of *Tnfsf14* and *S100a8* in NK cells may serve as a connection between innate and adaptive immunity during the development of the early immune response following BCG vaccination. However, this hypothesis requires further investigation.

*C5ar1* is one of the receptors of the complement anaphylatoxin C5a. This complement contributes to host defense against infection [83]. The activation of C5ar1 has been observed to stimulate various processes, including chemotaxis, granule enzyme release, intracellular calcium release, and superoxide anion production. In particular, the interaction of C5a initiates the accumulation of complement and phagocytic cells at sites of infection, as well as the recruitment of antigen-presenting cells to lymph nodes [84]. As evidenced by human single-cell RNA-seq data [85,86], C5ar1 expression within the bone marrow is predominantly observed in macrophages.

Considering that the site of infection was in the right flank of the mice, it is unsurprising that there was a reduction in C5ar1 expression in the bone marrow on day 3 after BCG vaccination. This may be attributed to two possible mechanisms: first, macrophages expressing this receptor may migrate to the site of inflammation; second, the expression of the receptor in macrophages may be suppressed to prevent the overactivation of the immune system.

*Csf2rb* encodes a common beta chain of type I cytokine receptors, which include receptors for GM-CSF, IL3, and IL5 [87]. These hematopoietic cytokines play an important role in inflammation. However, their precise functions are extremely diverse [88], and thus, no single conclusion can be drawn regarding their role in the development of trained immunity. As with the previously described proteins, the suppression of this gene’s expression may be related to the suppression of the early immune response at day 3 post-BCG vaccination.

Thus, we confirmed that local BCG immunization elicits early immune response of bone marrow and spleen innate immunity cells as early as 3 days following vaccination and provided valuable insights on the exact transcriptomic changes underlying these findings. However, the exact link between these findings and TRIM is yet to be further investigated. The insights obtained within our study can provide valuable information on the mechanisms underlying early immune response to BCG vaccination in mice.

## 5. Conclusions

In this study, we assessed the early response of mice to subcutaneous BCG vaccination by examining immune cell populations in the bone marrow and spleen, as well as the transcriptomic alterations occurring in neutrophils, monocytes, NK cells, and non-sorted bone marrow cells. An increase in the proportion of splenic NK cells and monocytes was observed following vaccination. In contrast, no alterations were identified in neutrophils, both in terms of the cell percentage and at the transcriptomic level, following BCG vaccination. Transcriptomic analysis revealed the enrichment in Gene Ontology terms related to the regulation of immune response in NK cells, monocytes, and unsorted bone marrow cells following BCG vaccination. Two NK cell-specific immune ligands (Tnfsf14 and S100a8) and two bone marrow-specific immune receptors (C5ar1 and Csf2rb) were identified. However, further research is required to elucidate the roles of the identified genes and gain a deeper understanding of the trained immunity phenomenon.

## Figures and Tables

**Table 1:** Data quality parameters for each sample.

Scheme	Total Number of Paired-End Reads, mln	Uniquely Mapped Reads, %	Assigned Reads, %
1. NK control	65,162,324	83.7	44.2
2. NK BCG	48,924 684	72.4	39.1
3. NK control	46,873,566	84.6	42.5
4. NK BCG	64,495,716	58.6	28.6
5. NK control	55,366,344	82.9	46.5
6. NK BCG	74,519,736	85.1	43.5
1. Neutrophil control	62,399,420	86.6	51.9
2. Neutrophil BCG	40,533,212	85.6	50.3
3. Neutrophil control	33,291,886	87.5	52.4
4. Neutrophil BCG	50,416,772	85.2	51.8
5. Neutrophil control	62,632,742	85.9	49.9
6. Neutrophil BCG	96,455,090	74.9	45.1
1. Monocyte control	60,950,058	83.2	52.5
2. Monocyte BCG	82,245,150	80.2	50.4
3. Monocyte control	43,105,930	79.2	52.2
4. Monocyte BCG	29,940,714	82.8	51.6
5. Monocyte control	55,015,316	83.6	52.7
6. Monocyte BCG	61,669,888	83.6	49.5
1. Bone marrow control	63,168,828	40.1	21.2
2. Bone marrow BCG	81,364,514	38.8	17.8
3. Bone marrow control	67,743,872	53.5	28.5
4. Bone marrow BCG	65,372,628	36.8	21.1
5. Bone marrow control	66,961,914	35.9	24.8
6. Bone marrow BCG	39,376,158	42.0	18.3

## Data Availability

Raw sequencing data and DESeq2-normalized counts have been deposited in NCBI’s Gene Expression Omnibus [28] and are accessible through GEO Series accession number GSE261448 (https://www.ncbi.nlm.nih.gov/geo/query/acc.cgi?acc=GSE261448, accessed on 30 October 2024). The full description of the data is also available in [29].

## Supplementary Materials

The following supporting information can be downloaded at https://www.mdpi.com/article/10.3390/cells13242043/s1: Figure S1: FACS quality control; Figure S2: The network analysis of enriched GO terms; Table S1: Deseq2 and Metascape results.
