# Supplementary figures and images for: The Cellular and Transcriptomic Early Innate Immune Response to BCG Vaccination in Mice
Source: Cells. 2024 Dec 11;13(24):2043. doi: 10.3390/cells13242043 (PMC11674076; doi:10.3390/cells13242043)

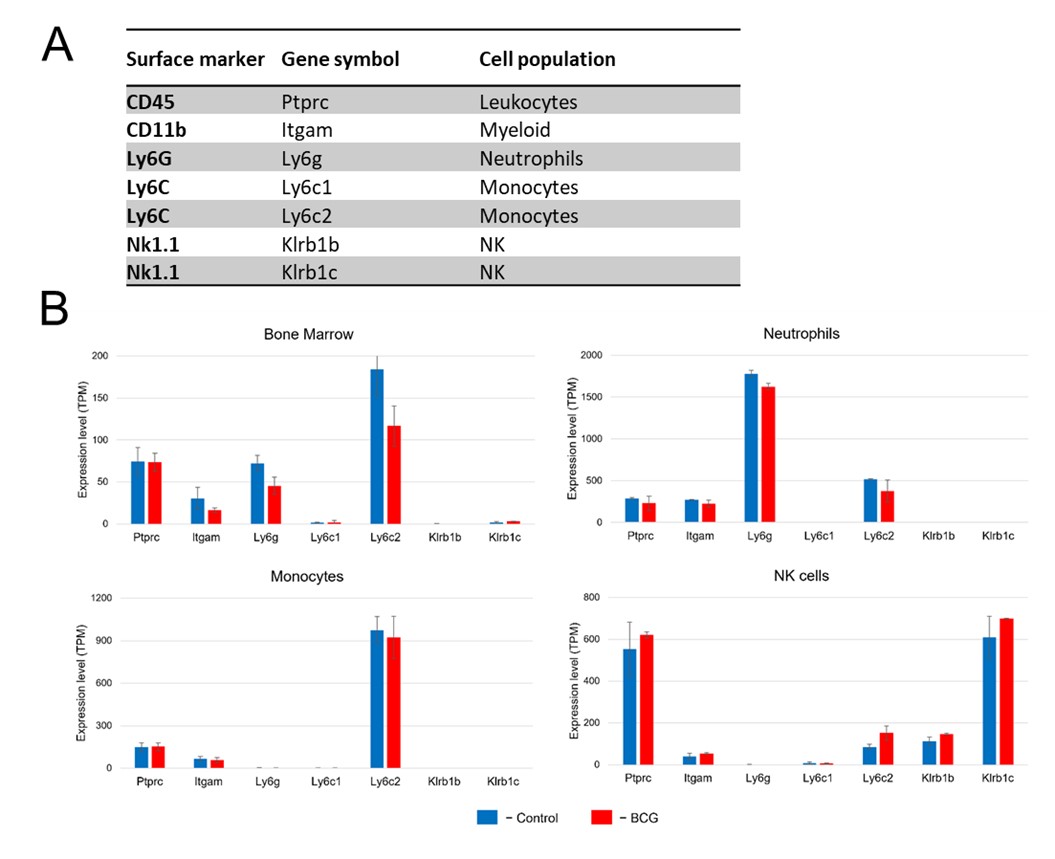

Supplement: Supplementary file 1 [file cells-13-02043-s001.zip › Figure S1.jpg]

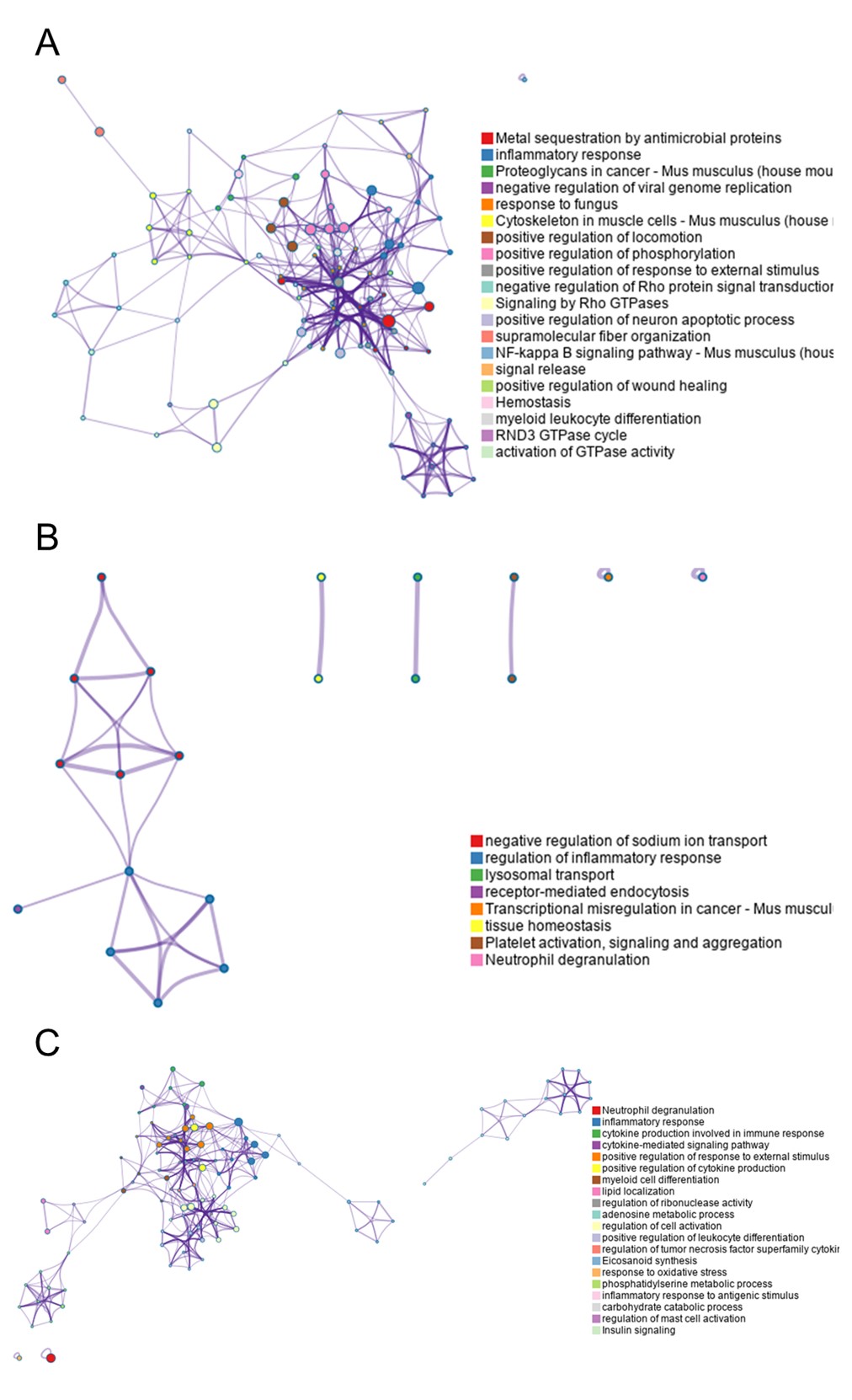

Supplement: Supplementary file 1 [file cells-13-02043-s001.zip › Figure S2.jpg]
